# Expression and function of aquaporin-1 in hyperoxia-exposed alveolar epithelial type II cells

**DOI:** 10.3892/etm.2014.1739

**Published:** 2014-05-28

**Authors:** QIU-YUE ZHANG, JIAN-HUA FU, XIN-DONG XUE

**Affiliations:** 1 Department of Pediatrics, Shengjing Hospital of China Medical University, Shenyang, Liaoning 110004, P.R. China; 2 Pediatrics Intensive Care Units, First Affiliated Hospital of Harbin Medical University, Harbin, Heilongjiang 150001, P.R. China

**Keywords:** alveolar epithelial type II cell, aquaporin-1, hyperoxia

## Abstract

The aim of the present study was to investigate water transport dysfunction in alveolar epithelial type II cells (AECII), which were exposed to hyperoxia, and to investigate the mechanism of pulmonary edema resulting from hyperoxic lung injury. The lung cells of newborn rats were isolated for primary cell culture and divided into control and experimental groups. The control and experimental group cells were placed into a normoxic incubator (oxygen volume fraction, 0.21) or hyperoxic incubator (oxygen volume fraction, 0.9), respectively. Twenty-four, 48 and 72 h after cell attachment, the gene transcription and protein expression levels of aquaporin-1 (AQP1) were detected via quantitative polymerase chain reaction and western blot analysis. Flow cytometry was conducted to detect the volume of the cells in the experimental and control groups. In the present study, it was identified that AQP1 expression and cell volume were greater in the experimental group when compared with the control group. Thus, hyperoxia may disturb the gene expression regulation of AQP1 in AECII, resulting in water transport dysfunction. This may be one of the mechanisms underlying pulmonary edema caused by hyperoxic lung injury.

## Introduction

Aquaporins (AQPs) are a family of hydrophobic transmembrane proteins that are selectively energized and impermeable to small molecules, which have been confirmed to exist in various entities, including bacteria, yeast and plants (1). Recently, various studies have demonstrated a complex network in the epithelium of the airway, which regulates water transportation, furthermore, the studies indicated that six types of AQPs are expressed in the lungs (2,3).

Numerous studies from the 1990s have identified that water is involved in the process of cell metabolism and achieves transmembrane transport via AQPs, which are highly selective for water molecules. AQPs, within various cells and tissues, are important in the immediate distribution of intra- and extracellular water under physiological and pathological conditions (4,5). Previous studies have shown that following birth, moisture was rapidly absorbed in the alveoli of mammalian embryos to enable a transition to spontaneous breathing, whilst AQP1 expression simultaneously increased in the lung tissue (6). AQP1 expression slowly increased in the fetal lung tissue and increased significantly as the fetus approached the pre-production period. Moreover, the expression exhibited time-phase variation shortly after birth (7). King *et al* (8) demonstrated that the intravenous injection of saline lead to peribronchiolar edema in healthy individuals, but no changes were observed in individuals with congenital absence of AQP1. A further study identified that the fluid transportation rate of the alveolar-capillary system decreased significantly within an AQP1 knock-out animal (9,10).

Previous studies have shown that numerous oxygen free radicals were produced in high oxygen environments and the integrity of the cell membrane was destroyed by the interaction of certain products of oxidative stress and inflammatory cytokines, which resulted in energy metabolism and cellular function disorders (11). Alveolar epithelial type II cells (AEC II) are the stem cells, which are critical for growth, development and wound repair processes in the lungs. Alveolar epithelial injury may be significant in the progression of lung injury in a high-oxygen atmosphere (12).

It has been observed that pulmonary edema is an early pathological change in lung tissue in a high-oxygen atmosphere (13); however, the mechanism of pulmonary edema at the cellular level remains unclear. Recent studies have demonstrated that AEC II was responsible for regulating fluid homeostasis in the lungs, however, this did not include regulation of surfactant secretion (14,15). It was hypothesized in the present study that problems with AEC II water permeability may exist in the early stages of hyperoxic lung injury due to fluid accumulation within the alveoli.

The present study primarily used cultured AEC II as the experimental model and applied quantitative PCR (qPCR) and western blot analysis to investigate the expression and functional changes in AQP1 under hyperoxic conditions. The aim was to explore the mechanism of pulmonary edema formation during lung injury with regard to the ability of the lungs to remove water.

## Materials and methods

### Isolation, culture and verification of AEC II

Culturing was performed as described by Dobbs *et al* (16). The present study was conducted in strict accordance with the recommendations laid out by the Guide for the Care and Use of Laboratory Animals of the National Institutes of Health. The animal use protocol was reviewed and approved by the Institutional Animal Care and Use Committee of Shengjing Hospital (Shenyang, China). Briefly, neonatal rats (<1 day old) were anesthetized with 10% chloral hydrate and the lung tissue was removed under sterile conditions. Subsequently, the lung tissue was washed using pre-cooled D-Hank’s solution and sectioned. Trypsin (1.5 ml; 0.25%; Merck KGaA, Darmstadt, Germany) was added and the tissue was digested in a 37°C water bath and agitated for 25–30 min. An equal quantity of Dulbecco’s modified Eagle’s medium (DMEM; HyClone, Logan, UT, USA) containing 10% fetal bovine serum (FBS; Clark, Seabrook, MD, USA) was added to terminate digestion, the tissue was filtered and centrifuged (143 × g) at 4°C for 5 min. Collagenase I (0.1%; Gibco-BRL, Carlsbad, CA, USA) was added, and the solution was digested for 15–20 min and centrifuged again (143 × g) at 4°C for 5 min. The cell pellets were resuspended in DMEM containing 10% FBS, transferred to anti-rat IgG-coated petri dishes (Santa Cruz Inc., Dallas, TX, USA) and incubated at 37°C for 15 min to purify the cells. Immunofluorescence of surfactant protein (SP)-C (a specific marker of AECII cells) and transmission electron microscopy (TEM; Olympus, Tokyo, Japan) were used to verify the AEC II cells, in addition, the viability of cells was determined by trypan blue staining. The purified cells were transferred to a 6-well plate and cultured for 12 h. After washing with PBS, the cells were fixed by 4% paraformaldehyde for 30 min, and then blocked by 10% FBS. SP-C antibody was added at 4°C for 12 h followed by adding FITC-labeled secondary antibody. In addition, the cellular nuclei were stained with DAPI, and subjected to fluorescence microscopy for observation.

### Groups and methods

The medium was changed following cell attachment. The cells were randomly divided into experimental and control groups and placed in a high oxygen (hyperoxic) incubator (oxygen volume fraction, 0.9) or a regular (normoxic) incubator (oxygen volume fraction, 0.21), respectively. The cells were collected after 24, 48 and 72 h of culture and stored at −80°C until they were assayed.

### qPCR

Total RNA was extracted from frozen samples and cDNA was synthesized via a reverse-transcription (RT) reaction. The PCR primers were designed using Primer Premier 5.0 (Premier Biosoft Inc., Paolo Alto, CA, USA) according to the rat AQP1 cDNA sequence, which was obtained from GenBank^®^ and the cDNA was synthesized by Sun Biotech Co., Ltd. (Beijing, China). The AQP1 primer sequence was as follows: Forward: 5′-ACCCGCAACTTCTCAAAC-3′ and reverse: 5′-CAGGTCATACTCCTCCACTT-3′. PCR was performed resulting in a final volume of 25 μl cDNA using a SYBR green PCR master mix reagent kit (USB, Cleveland, OH, USA). A qPCR device (Exicycler^™^ 96, Bioneer Company, Daejeon, South Korea) was used for quantitative analysis of the PCR product and the PCR reaction conditions were: 95°C for 10 min, 95°C for 10 sec and 60°C for 40 sec over a total of 40 cycles. The results were derived from the comparative threshold cycle (Ct) method and normalized by glyceraldehyde-3-phosphate dehydrogenase (GAPDH) as an internal control. The ΔΔCt method was applied to analyze the relative AQP1 gene expression level observed in the different samples.

### Western blot analysis

Protein was extracted from frozen lung tissue samples and homogenized in radio-immunoprecipitation assay lysis buffer with phenylmethanesulfonylfluoride. The protein was quantified and separated via SDS-PAGE gel electrophoresis and transferred to a cellulose membrane (Biyuntian, Jiangsu, China). Non-fat milk (5%) was used to block the membrane for 2 h at room temperature and a mouse anti-rat AQP1 primary antibody (1:500; Santa Cruz Biotechnology, Inc., Santa Cruz, CA, USA) was added, followed by incubation at 37°C for 1 h. Alkaline phosphatase conjugated goat anti-mouse secondary antibody (1:10,000; Zhongshan Jinqiao Biotechnology Co., Ltd., Beijing, China) was added, incubated at 37°C for 40 min and developed using an electrochemiluminescence kit (Milipore, Billerica, MA, USA). The film was digitized via an imaging system (Olympus) and the relative gray values were calculated with the following formula: Relative gray value=gray value of target band/gray value of internal control (GAPDH). Where, a higher relative gray value indicated a higher protein content.

### Measurement of cell volume

Following 24, 48 and 72 h of culture the primary cultured AEC II cells were washed with phosphate-buffered saline (PBS) and digested in 0.25% trypsin for 10 min. An equal quantity of DMEM containing 10% FBS was added to terminate digestion, the cells were centrifuged at 143 × g for 5 min, washed twice with PBS and resuspended into a single cell suspension with a final concentration of 10^9^ cells/ml. The 0.5 ml cell suspension was loaded into a flow cytometer (FACSCalibur^™^, BD Biosciences, Franklin Lakes, NJ, USA), 10,000 cells were detected and the average intensity of the forward angle light scatter (FALS) was calculated (17). Briefly, when cells are sprayed out from the nozzle of the flow cytometer in a single line, exposure to the laser causes forward scattering of the light. The intensity of the FALS is proportional to cell size, the larger the cell volume, the higher the intensity of the scattered light. Therefore, the average intensity of FALS was used as an indicator of cell volume.

### Statistical analysis

SPSS 13.0 software (SPSS Inc., Chicago, IL, USA) was used for statistical analysis and data were presented as the mean ± standard deviation. Two-factor analysis of variance was applied and an L-matrix program (programmed analysis of matrix) enabled comparisons between the two groups. P<0.05 was considered to indicate a statistically significant difference.

## Results

### Identification of AEC II

**Figure 1:**
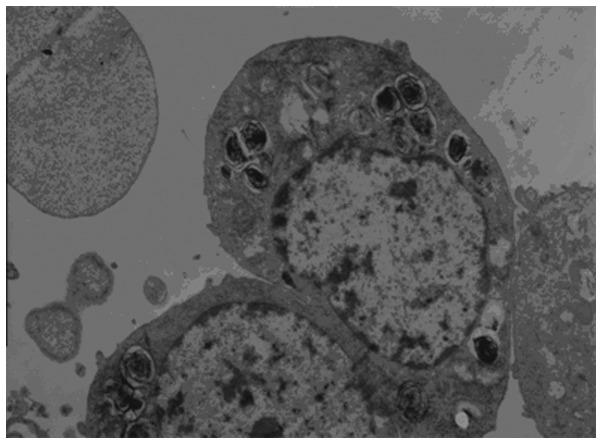
AEC II observed via TEM (magnification, ×5,000). TEM was conducted to verify AEC II. The characteristic lamellar bodies were detected in the AEC II. AEC II, alveolar epithelial type II cells; TEM, transmission electron microscopy.

The characteristic lamellar bodies were detected in the AEC II via TEM and appeared to be arranged as concentric round, or parallel layered structures. In addition, mitochondria, endoplasmic reticulum and Golgi apparatus were detected, and microvilli were observed at the luminal side of the cell (Fig. 1).

**Figure 2:**
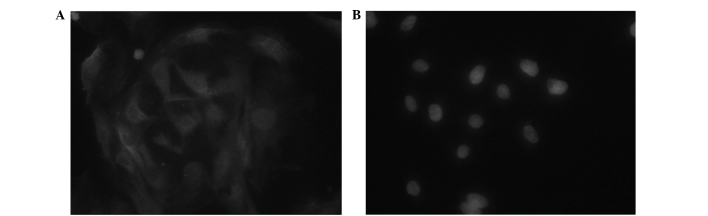
AEC II stained immunofluorescence stain of FITC (magnification, ×400). (A) AEC II cells were incubated with SP-C (an AEC II-specific pulmonary surfactant) monoclonal antibody. (B) The omission of primary antibodies served as a negative control. AEC II, alveolar epithelial type II cells.

AEC II secretes pulmonary surfactants, such as SP-A, -B, -C and -D, of which SP-C is the only AEC II-specific surfactant, therefore, it can be used to identify AEC II (8). SP-C immunofluorescence staining detected fluorescein isothiocyanate-green fluorescence, which was located in the cytoplasm and the AEC II cells exhibited positive expression of SP-C (Fig. 2). The cell purity was 92.5±3.28% and a trypan blue dye exclusion method revealed a cell viability of 95±1.06%.

### AQP1 gene

**Figure 3:**
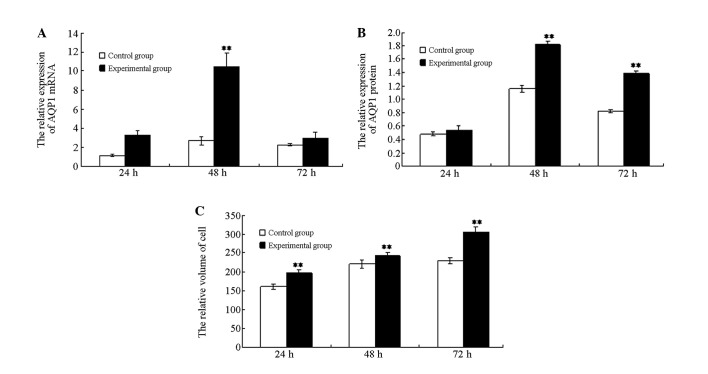
Expression and function of AQP1 in hyperoxia-exposed AEC II. (A) The dynamic change in the relative expression of AQP1 mRNA in hyperoxia-exposed AEC II. The relative expression of the AQP1 mRNA from the cells in the control and experimental group was analyzed following 24, 48 and 72 h of culture using quantitative polymerase chain reaction. (B) The protein expression of AQP1 in AEC II; cells from the control and experimental group were collected following 24, 48 and 72 h of culture. Western blot analysis was conducted to detect the protein expression of AQP1 in the AEC II. (C) The change in cell volume in the hyperoxia-exposed AEC II. The volume of cells from the control and experimental group was assessed by flow cytometry following 24, 48 and 72 h of culture. Flow cytometry data are presented as histograms of the average intensity of the forward angle light scatter as an indicator of the cell volume. The results are representitive of three independent experiments. ^**^P<0.01 compared with the control group. AQP1, aquaporin-1; AEC II, alveolar epithelial type II cells.

qPCR analysis demonstrated that AQP1 mRNA expression in the experimental group cells was significantly increased at 48 h following hyperoxia when compared with the cells of the control group (P<0.01), while no significant differences were detected at 24 and 72 h between the two groups (P>0.05). AQP1 mRNA expression of the experimental group increased initially and subsequently decreased, which was a statistically significant change (P<0.01; Fig. 3A).

### AQP1 protein

**Figure 4:**
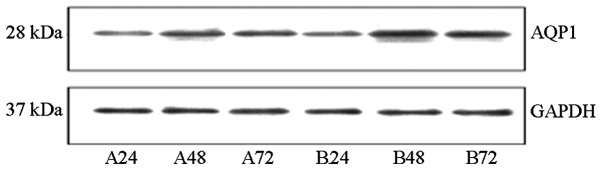
The protein expression of AQP1 in AEC II. The representative western blot analysis examples of the AQP1 expression band density of each group were normalized to the total internal control (GAPDH) and expressed as a percentage of the control. A24, A48, and A72 are control groups for 24, 48 and 72 h, respectively. B24, B48, and B72 are hyperoxia-exposed group for 24, 48 and 72 h, respectively. AQP1, aquaporin-1; AEC II, alveolar epithelial type II cells; GAPDH, glyceraldehyde-3-phosphate dehydrogenase.

Western blot analysis indicated that at 48 and 72 h, the AQP1 protein content in the cells of the experimental group was significantly higher than that of the control group (P<0.01). However, the AQP1 protein level was not significantly different between the two groups at 24 h. The AQP1 protein expression in the experimental group initially increased and subsequently decreased, which was a statistically significant change (P<0.01; Fig. 3B). These data were consistent with the fluorescence qPCR results (Fig. 4).

### Cell volume

**Figure 5:**
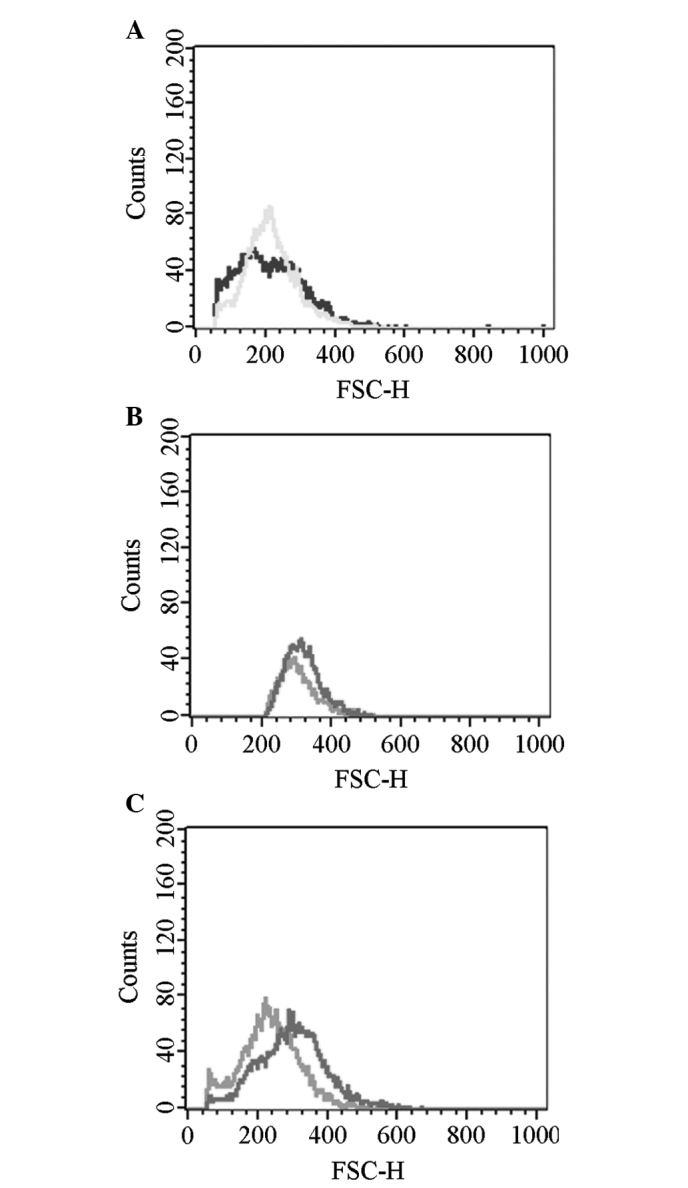
Change in cell volume in the hyperoxia-exposed alveolar epithelial type II cells following culturing for 24, 48 and 72 h represented by plots of the average FSC intensity of the fluorescence-activated cell sorting analyses. (A) Counts at 24 h: Dark, control group; light, experimental group. (B) Counts at 48 h: Light, control group; dark, experimental group. (C) Counts at 72 h: Light, control group; dark, experimental group. FSC-H, forward scatter height.

The cell volume of the experimental group (hyperoxia) was markedly greater than the control group (normoxia; P<0.01). An intra-group comparison within the experimental group indicated that the cell volume gradually increased with prolonged hyperoxia exposure and the differences were identified to be statistically significant (P<0.01; Figs. 3C and 5).

## Discussion

Yue *et al* (18) demonstrated that the alveolar membrane intervals were widened and edema were observed in the hyperoxic group. Furthermore, lanthanum nitrates were shown to be scattered in the cellular junction. Moreover, a varying quantity of lanthanum particles appeared in AEC II cells and in the mesenchyma of the alveolar septum, which indicated that the increased permeability of the alveolar epithelial cells had contributed to pulmonary edema (18). However, the pathogenic mechanisms for hyperoxic pulmonary edema remain unclear. Studies have shown that AQP1 may be important in lung fluid transportation, as well as in the pathogenesis of pulmonary edema (19).

The aim of the present study was to test the hypothesis that imbalanced AQP1 expression may be one of the pathogenic mechanisms for hyperoxic lung injury and pulmonary edema at the cellular level. AQP1 transcription and protein expression in the cells of the hyperoxia group was observed to be significantly increased compared with the cells of the control group.

Among the various lung injuries investigated, there was a reduction in AQP1 expression or activity, and AQP1 and AQP4 mRNA expression was downregulated in allergen-induced mouse models of asthma (20). Previous studies have identified that in hyperoxic lung injury-induced pulmonary edema mice models, the expression level of AQP1 in lung tissues was significantly downregulated (21). Lipopolysaccharide-induced acute lung injury significantly impaired alveolar fluid clearance; however, following administration of dopamine, this symptom was relieved (via upregulation of AQP1 and AQP5 expression), which may have further promoted the reabsorption of alveolar fluids (22). *Staphylococcus aureus* and its major pathogenic factor, α-hemolysin, have been shown to significantly affect AQP1 expression (23). Furthermore, previous studies have indicated that viral infections and hypoxia result in pulmonary edema that is accompanied by the downregulation of AQP1 expression (24,25). Decreased lung AQP1 and AQP5 expression may be associated with pulmonary edema development and increased severity of lung injury and pulmonary edema, which may provide an additional mechanism for pancreatitis-associated lung injury (26).

However, certain studies indicated that AQP1 expression was increased during lung injuries. For example, Lai *et al* (27) demonstrated that inflammatory factors were able to promote AQP1 expression in a cell model. In another study, Li *et al* (28) used results from RT-PCR and western blot analysis to show that the intratracheal administration of seawater upregulated the mRNA and protein levels of AQP1 and AQP5 in the lung tissues. Song *et al* (29) reported that AQPs were not required for the physiological clearance of lung water in neonatal or adult lungs or for the accumulation of extravascular lung water in an injured lung. The results of the present study were consistent with those of Li *et al* and Song *et al*. It was hypothesized that the widened alveolar intervals, the infiltration of inflammatory cells into the mesenchyma of the lung, congestion, hyperplasia and/or reconstruction of the pulmonary vasculature of the normal lung tissues, may have increased the thickness of the membrane between the alveolar and vascular cavity, which may have affected fluid transport. Thus, the osmotic pressures were altered, which significantly influenced electrolyte metabolism and lung ventilation. We found that the AQP1 under hyperoxic conditions was increased at transcription and protein level. This suggests that the enhanced water transportation is one of the compensatory mechanisms for improving body internal environment. Furthermore, over a prolonged time period in a high-oxygen atmosphere, the lung injury was gradually aggravated, which may have resulted in decreased expression levels of AQP1 mRNA and protein.

Tsai *et al* (30) hypothesized that inducible NO synthase was enhanced by the high oxygen environment, either directly or via signaling of the NF-κB transduction pathway, which may have induced excessive inflammatory mediator expression, such as tumor necrosis factor (TNF)-α, interleukin (IL)-β, prostaglandin E_2_ (PGE_2_) and matrix metalloproteinases (MMPs). These inflammatory mediators may have led to an increase in endothelial permeability, emergence of edema and enhanced levels of transcription. This was consistent with a previous study where excessive inflammatory mediator expression (TNF-α, IL-β, PGE_2_ and MMPs) were induced, which resulted in the increased permeability of the endothelial cells and edema (31). A large number of oxygen free radicals may activate the signaling pathways of mitogen-activated protein kinases, including the three subunits, extracellular signal-regulated kinase (ERK), P38 and c-Jun amino-terminal kinase (JNK). It has been identified that astrocytes *in vitro* inhibited the expression of AQP1 via the JNK pathway (32). However, the ERK pathway activated the expression of AQP1 and AQP5 in hypertonic conditions (33). Furthermore, activation of the P38 signaling pathway upregulated the expression of AQP5 in lung cancer tissue (34).

Thus, it was hypothesized that, as a result of varied cell volume during water transportation, plasmalemma expansion or contraction occurs, which may activate the mechanically gated ion channels or mechanical receptors on the plasmalemma. Subsequently, specific signal transductors were activated that adjusted the transportation of cellular fluids (35), which may lead to lung edema during acute hyperoxic lung injury.

In our study, the cell volume was gradually increased under hyperoxic conditions, but the AQP1 expression was not induced at 72 h when compared with that in control group. This, therefore, indicated that AQP1 may not be the only mechanism of fluid transport in the cell membrane of AEC II, with other fluid-balance transport systems present, such as sodium channels and sodium-potassium ATPase enzymes.

In conclusion, there are few studies regarding the mechanisms of fluid clearance in hyperoxic lung injury and lung edema, thus, there is currently no definitive conclusion. The present study indicated that AQP1 water transport dysfunction may be a predominant cause of pulmonary edema in acute lung injury, however, further studies are required to provide further confirmation.
